# Chytrid fungi infecting Arctic microphytobenthic communities under varying salinity conditions

**DOI:** 10.1038/s41598-024-77202-2

**Published:** 2024-10-28

**Authors:** Doris Ilicic, Jason Woodhouse, Ulf Karsten, Katherina Schimani, Jonas Zimmermann, Hans-Peter Grossart

**Affiliations:** 1 Department of Plankton and Microbial Ecology, Leibniz Institute of Freshwater Ecology and Inland Fisheries, Neuglobsow, Germany; 2 Department of Applied Ecology and Phycology, Institute of Biological Sciences, University of Rostock, Rostock, Germany; 3 Department of Maritime Systems, Interdisciplinary Faculty, University of Rostock, Rostock, Germany; 4 Botanic Garden and Botanical Museum Berlin, Freie Universität Berlin, Berlin, Germany; 5 Institute of Biochemistry and Biology, University of Potsdam, Potsdam, Germany

**Keywords:** Chytrids, Arctic, Fungal parasites, Microphytobenthos, Zoosporic fungi, Microbiology, Ecology, Microbial ecology

## Abstract

This study aimed to investigate the presence and diversity of fungal parasites in Arctic coastal microphytobenthic communities. These communities represent a key component in the functioning of Arctic trophic food webs. Fungal parasites, particularly Chytridiomycota (chytrids), play significant roles by controlling microalgal bloom events, impacting genetic diversity, modifying microbial interactions, and accelerating nutrient and energy transfer to higher trophic levels. In the context of rapid Arctic warming and increased glacier meltwater, which significantly affects these communities, we used high-throughput sequencing to explore fungal community composition. Our results show that chytrids dominate fungal communities in Arctic benthic habitats and that the overall fungal diversity is primarily influenced by the salinity gradient. Chytrid representation is positively correlated with the presence of potential benthic diatom (*Surirella*, *Nitzschia*, *Navicula*) and green algae (Ulvophyceae) hosts, while microscopic observations provide further evidence for the presence of active chytrid infections.

**Supplementary Information:**

The online version contains supplementary material available at 10.1038/s41598-024-77202-2.

## Introduction

Arctic waters are rich in life and include numerous habitats and substrates that are known, or assumed, to host unique species. In recent decades, this region has experienced significant changes in its environmental factors attributed to global change, resulting in a substantial decline in sea-ice extent and duration of the sea-ice season^1^. Arctic aquatic ecosystems are experiencing localized shifts characterized by elevated water temperatures and modifications in light penetration, dust deposition, sediment load, and salinity levels due to intensified glacial melting and subsequent terrestrial runoff^2^. Particularly susceptible to these changes are slow-moving or sedentary benthic communities in shallow coastal areas, which experience significant impacts on their species richness, diversity, and function^3,4^.

Microphytobenthic communities are particularly important components of many Arctic benthic habitats^5–7^. Generally dominated by pennate diatoms, they hold important ecological roles as their productivity exceeds that of pelagic microalgae and can account for more than 90% of total production^8^. As such, they provide energy supply to Arctic food webs^9^, serve as a major food source for benthic suspension- or deposit-feeders^10^ and play important roles in the global carbon cycle^11^. Additionally, these protists have been known as preferential hosts for fungal parasites, such as species within the phylum Chytridiomycota (chytrids)^12^.

Chytrids belong to the early diverging lineages of so-called “dark matter” fungi^13^ and are ubiquitous and highly abundant in aquatic ecosystems^14–16^. They exhibit a wide range of consumer strategies, ranging from obligate saprotrophs to obligate parasites^17^. As such, they are decomposers of autochthonous and allochthonous organic matter (e.g. pollen grains, zooplankton exuviae)^18,19^ and lethal parasites of many microalgal species^17^. When it comes to parasitic chytrids, different species show different infection strategies^17^; they are either generalists, capable of infecting many host species, or specialists that infect only one specific host^20–22^. General life cycle of chytrid parasites involves free-living, motile, uniflagellated zoospore that serves as a primary mechanism for dispersal, and a sporangium, a structure that is attached to a host cell surface via rhizoids and in which new zoospores are formed^23,24^. Parasitic chytrids can control microalgal population dynamics by causing delay or even suppressing bloom events^25,26^. Through selective parasitism, they alter intra- and interspecific competition, affecting coexistence and promoting genetic diversity in host populations^27,28^. Moreover, they modify microbial interactions in aquatic food webs. Bypassing microbial loop through fungal shunt^29^ and mycoloop^30^ they serve as a trophic link between primary and secondary producers^31–33^.

Although the research on chytrids in the Arctic is still in its infancy, recent studies using high-throughput sequencing technologies have shown their prevalence in fungal communities across various habitats including lakes^34,35^, streams and ponds^36^, seawater^37^, sea-ice^38–40^, and sea-floor sediments^41^. However, we are still lacking the understanding of parasitic chytrids, encompassing their diversity, seasonal patterns, infection strategies, and the factors driving parasitism^13^. Some studies have reported microscopical observations of parasitic interactions. For instance, Fiołka et al.^42^ observed chytrid infections on snow and glacier algae *Ancylonema nordenskioeldii* and *Sanguina nivaloides* at Longyearbreen and Foxfonna glaciers on Svalbard, and Kobayashi et al.^43^ reported chytrid infection on *A. nordenskioeldii*, *A. alaskana*, *S. nivaloides* and *Chloromonas* sp. in cryoconite holes in Alaska. Moreover, Hassett and Gradinger^41^ conducted a study indicating the seasonal prevalence of chytrid parasitism in sea-ice and sea-floor sediment in Barrow, Alaska. They observed that chytrid populations exhibited seasonal fluctuations, with peak abundances aligning with the onset of the algal spring bloom. However, benthic diatoms of the Arctic shallow coastal zones remain largely understudied and their biotic interactions with chytrid parasites and their implications for food web dynamics, alongside the influence of ongoing global change on these interactions, predominantly unknown.

**Fig. 1:**
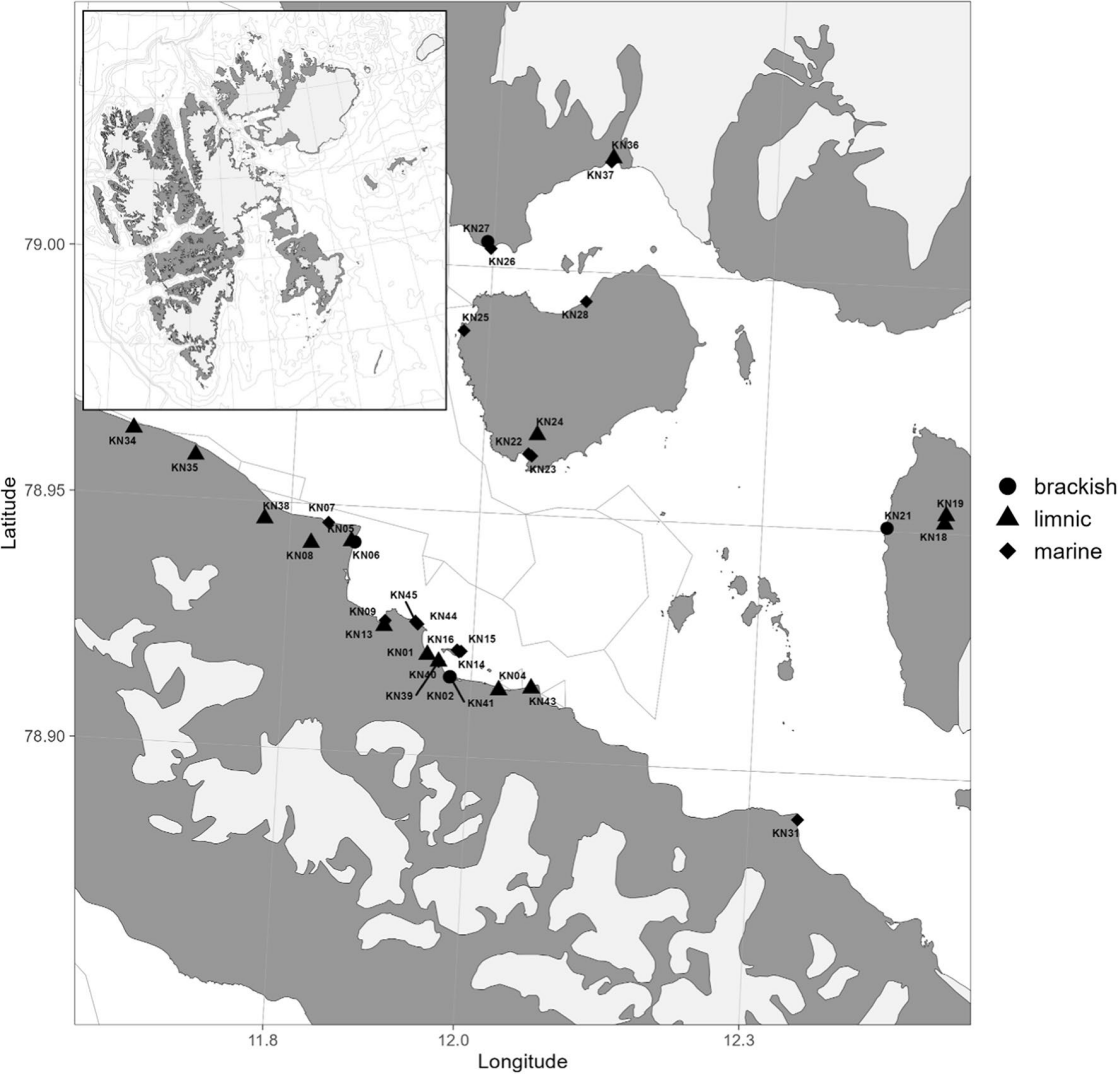
Map of Kongsfjorden (Svalbard, Norway). Sampling sites including limnic, brackish and marine shallow waters.

In this study, we investigated the presence and diversity of chytrids in microphytobenthic communities of Arctic shallow coastal waters (Fig. 1). Specifically, we aimed to investigate parasitic lifestyle of chytrids and the distribution of chytrid parasites in relation to abiotic parameters such as temperature and salinity gradient, as well as the composition of the wider eukaryotic community, including putative hosts. We used both the eukaryotic general SSU rDNA and fungal-specific LSU region, coupled with high-throughput Illumina sequencing, to characterize microphytobenthic communities and assess the diversity and distribution of parasitic chytrid and putative host sequences. By integrating microscopy, we verified parasitic interactions, identified host-parasite pairs, and determined the prevalence of infections within the sampled community.

## Results

### Environmental factors

**Fig. 2:**
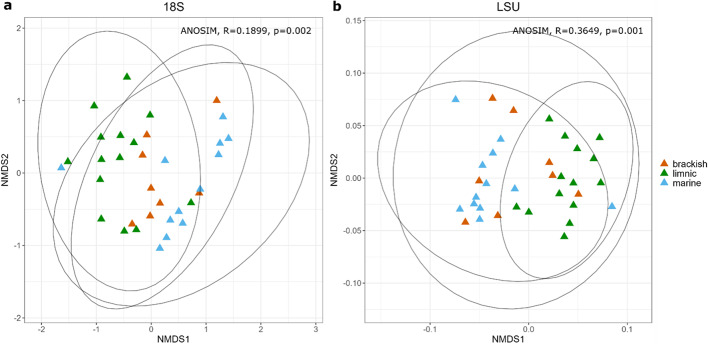
Eukaryotic and fungal community structure differences related to salinity. NMDS multivariate clustering of (**a**) wider eukaryotic (SSU) and (**b**) fungal communities (LSU) according to water type and computed by the Bray–Curtis dissimilarity index.

**Fig. 3:**
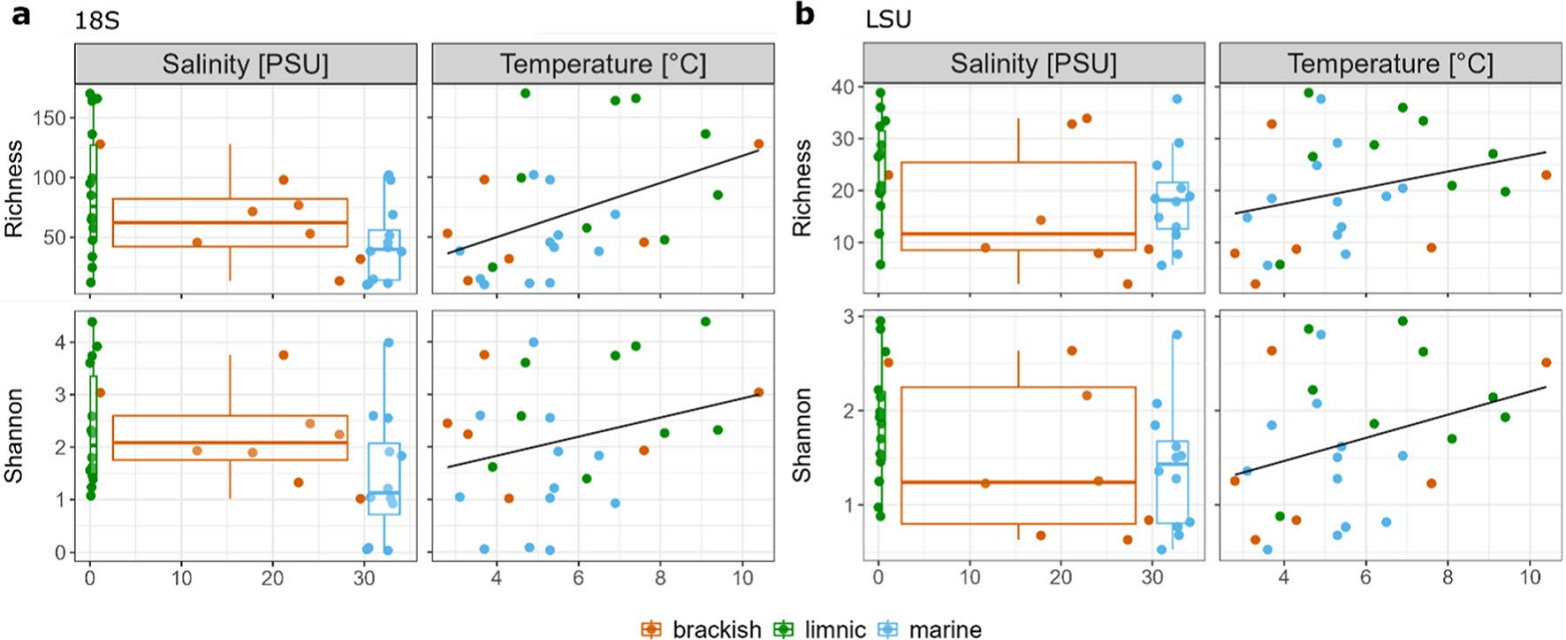
Effects of environmental parameters on eukaryotic and fungal community structure. Relationship between species richness and Shannon diversity index and salinity (PSU) and temperature (°C) in (**a**) wider eukaryotic (SSU) and (**b**) fungal communities (LSU). Data are fitted to linear regressions and significant relationships assessed using Pearson correlation.

Non-dimensional metric scaling (NMDS) ordination analysis comparing the SSU community structure showed significant differences in communities between marine, limnic and brackish habitats (ANOSIM, *R* = 0.1899, *p* = 0.002), where marine and limnic samples formed distinct clusters (*p* = 0.003) (Fig. 2A). Fungal communities exhibited the same pattern (ANOSIM, *R* = 0.3649, *p* = 0.001) and significant differences were observed between marine and limnic habitats (*p* = 0.003) (Fig. 2B). To examine the relationship between community dynamics and environmental factors in more detail, Pearson’s correlation and simple linear regression analysis were performed on alpha diversity measures such as species’ richness and Shannon diversity indices. The results revealed that salinity significantly impacted species’ richness only in fungal communities (Kruskal–Wallis test, *p* = 0.016), whereas a significant impact on the Shannon diversity was observed for both wider eukaryotic (Kruskal–Wallis test, *p* = 0.014) and fungal communities (Kruskal–Wallis test, *p* = 0.02). The wider eukaryotic community in marine samples exhibited a significantly lower diversity with less species observed compared to brackish and limnic samples (pairwise Wilcoxon test, *p* = 0.04 and *p* = 0.02, respectively) (Fig. 3A). Fungal communities exhibited a similar pattern, with significantly less diverse communities observed in marine compared to the limnic samples (pairwise Wilcoxon test, *p* = 0.02), while the number of species detected was significantly higher in limnic compared to the brackish and marine samples (pairwise Wilcoxon test, *p* = 0.04 and *p* = 0.03, respectively) (Fig. 3B). Simple linear regression showed that temperature significantly influenced species’ richness in wider eukaryotic communities, explaining 21% of variance (R2 = 0.2105, *p* = 0.016), whereas no significance was observed for the diversity (R2 = 0.087, *p* > 0.05) (Fig. 3A). Conversely, temperature explained only 8.6% of variance in species’ richness and 9.2% of variance in diversity in fungal communities, with no statistical significance observed (R2 = 0.086, *p* > 0.05 and R2 = 0.092, *p* > 0.05, respectively) (Fig. 3B). Species’ richness and Shannon diversity indices were positively correlated with temperature in both datasets, but negatively correlated with salinity (Supplementary Material Tables 2 and 3).

### Diversity and community composition

**Fig. 4:**
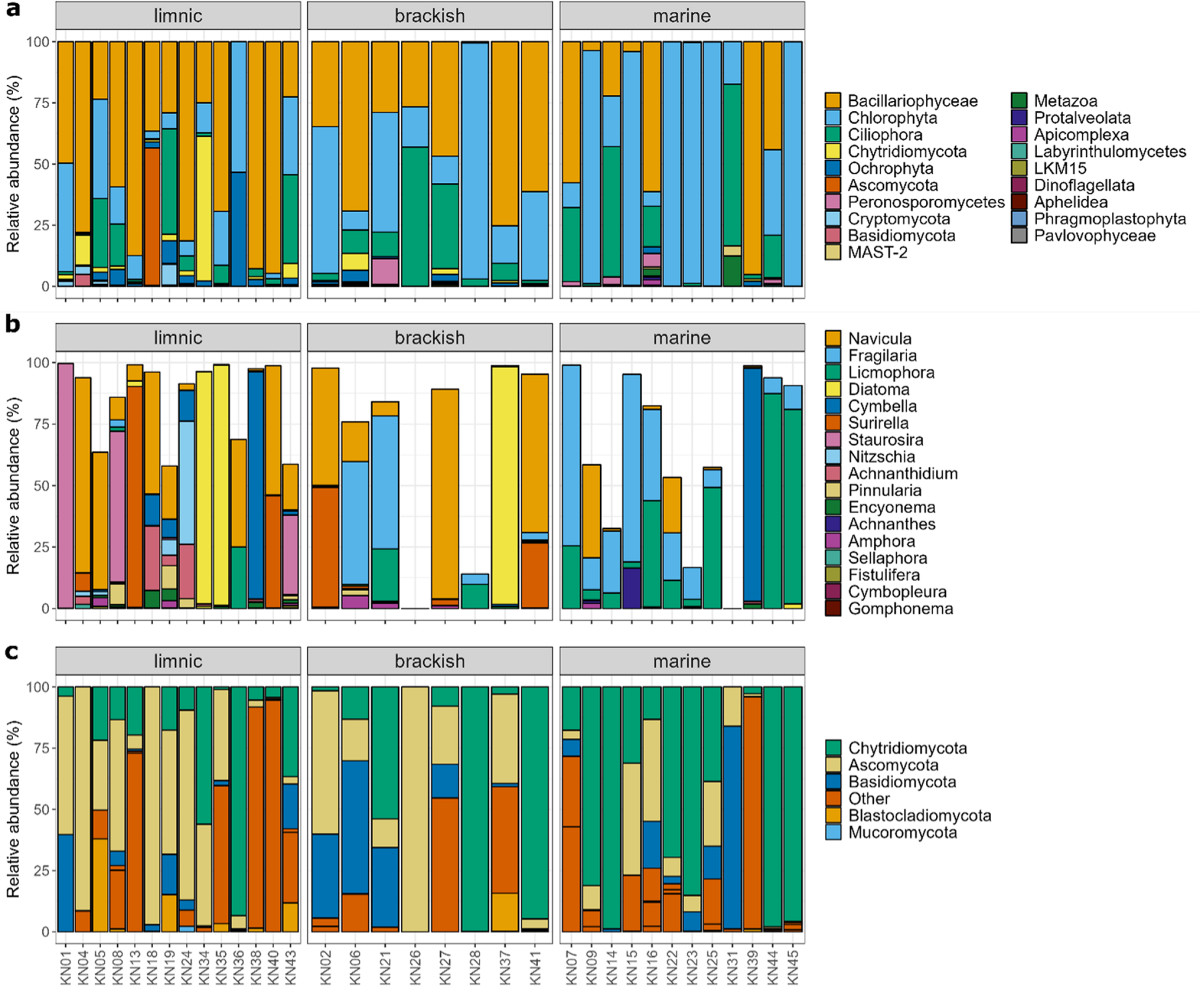
Eukaryotic and fungal community composition between three different water types of the Arctic benthic habitats. (**a**) Eukaryotic community composition (SSU) at the phylum level, (**b**) OTU abundances of diatom genera relative to the total diatom community (Bacillariophyta), (**c**) fungal community composition (LSU) at the phylum level.

Of the 446 OTUs generated in the SSU dataset, 73 belonged to Bacillariophyceae, 72 to Chlorophyta, 54 to Ochrophyta, and the rest belonged to Fungi, Metazoa, Aphelida, Cryptomycota (Rozellomycota) and supergroup SAR (Fig. 4A). Bacillariophyceae (diatoms) consisted primarily of pennate diatoms and their proportions differed between sampled water types (Fig. 4B). *Navicula* was predominantly found in brackish and freshwater samples, while *Fragilaria* and *Licmophora* dominated among diatoms in marine samples. In addition, 53 OTUs in the SSU dataset were classified as Fungi (4 Ascomycota, 23 Chytridiomycota, 23 Basidiomycota, 3 Neocallimastigomycota). However, more variable and less conserved large subunit (LSU) 28S rRNA gene allows for better discrimination between different fungal taxa and is thus typically used in fungal taxonomy and phylogenetics.

**Fig. 5:**
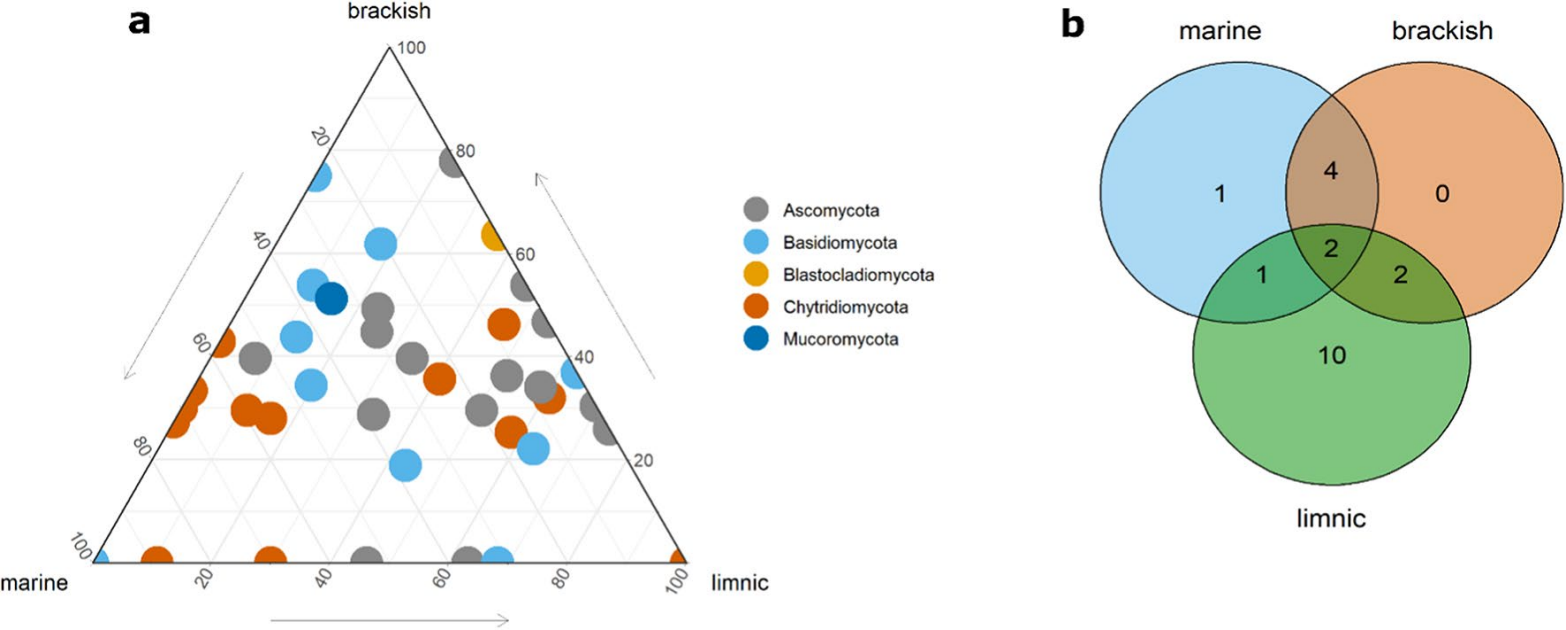
Overlap of fungal OTUs recovered from sampled water types. (**a**) Ternary plot showing relative abundance of fungal OTUs in the LSU dataset. Each point represents an OTU. The position represents the relative abundance of the OTU with respect to each water type. (**b**) Venn diagram showing the overlap of chytrid OTUs among the three different water types.

Our LSU dataset consisted of 151 OTUs in total, of which Ascomycota and Chytridiomycota were the most abundant fungal taxa with 31 and 18 OTUs, respectively (Fig. 4C). Other fungal OTUs belonged to Basidiomycota (17), Blastocladiomycota (5), Mucoromycota (1), or were classified as “unknown” (51). The rest of the OTUs in the LSU dataset classified as taxa belonging to the SAR supergroup. Chytridiomycota and Blastocladiomycota were represented by orders Rhizophydiales, Lobulomycetales, Chytridiales, Mesochytriales and Blastocladiales, and 9 out of 18 OTUs within Chytridiomycota were classified as “unknown”. Proportions of fungal OTUs and their abundances differed between sampled water types. Ascomycota were most abundant in limnic samples, while Chytridiomycota dominated in brackish and marine samples. Moreover, many OTUs were shared between limnic, brackish and marine samples (Fig. 5A). Out of 18 Chytridiomycota OTUs, 10 were specific for limnic and 1 for marine habitat (Fig. 5B). However, the highest overall abundance of Chytridiomycota OTUs was found in marine habitats. This indicates that, while only one Chytridiomycota OTU was specific to the marine habitat, other OTUs detected were also present in either limnic or brackish habitats, with 2 OTUs being shared across all three water types.

**Fig. 6:**
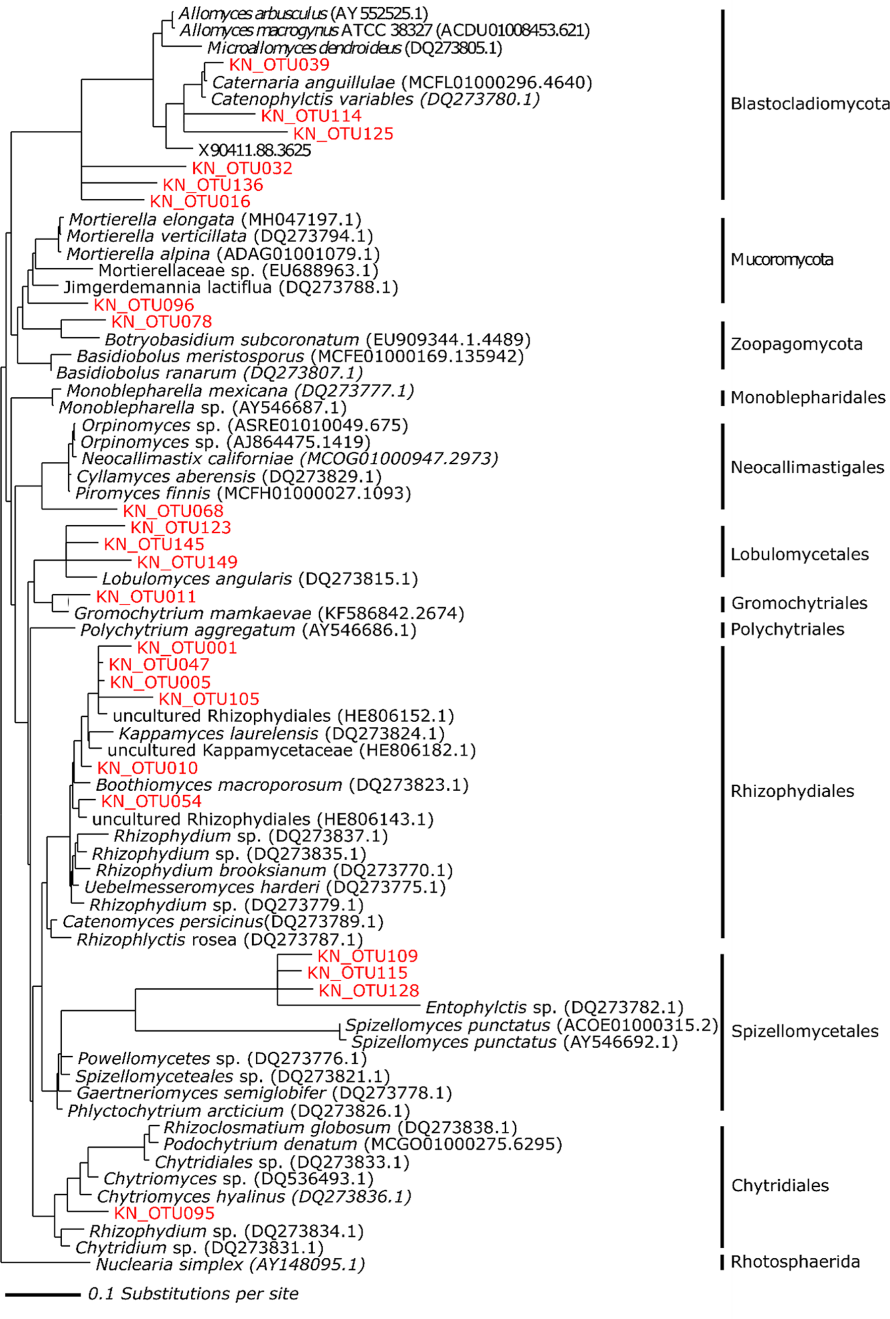
Phylogenetic classification of chytrid OTUs detected in the LSU dataset. Phylogenetic tree was built using the Maximum Likelihood method. Sequences obtained in this study are colored in red.

Phylogenetic analysis provided a deeper insight into the unknown proportion of Chytridiomycota sequences (Fig. 6). OTU095 confirmed its position within Chytridiales clade. OTU109, OTU115 and OTU128, primarily assigned as Rhizophydiales, formed a separate cluster within the Spizellomycetales clade. Six OTUs clustered within Rhizophydiales, 3 within Lobulomycetales and 1 within Neocallimastigales clade. Additionally, one OTU clustered within Zoopagomycota and one within Mucoromycota clade.

### Host-parasite interactions

The prevalence of chytrid infections on microalgal cells ranged from 0 to 9.5% per sample (Supplementary Material Fig. S1). Highest infection prevalence was observed in marine samples, where infections were predominantly found on green algae (*Ulothrix*) filaments (Supplementary Material Fig. S2). Most of the infected filaments had multiple sporangia attached, whereas in some samples only one sporangium per filament was observed. In limnic samples, infections were observed mainly on pennate diatoms (e.g. *Navicula*, *Nitzschia*), which dominated microphytobenthic community (Supplementary Material Fig. S3). In samples KN13 and KN40, *Surirella* cells accounted for 31% and 28% of the total microalgal cells, based on microscopic cell counts. In both cases, it was the only infected taxa in the sample, with an infection prevalence of 16.8% and 3.1%, respectively. This corresponds to an overall infection prevalence of 6.1% and 0.9% when considering the total number of cells in the sample. Maximum number of sporangia per infected cell was two. Moreover, one (KN02) out of three brackish samples contained infected cells, where fungal sporangia were detected on pennate diatoms, with the infection prevalence of 4%. In samples KN37 and KN28, fungal sporangia were observed on green algae filaments, with the infection prevalence of 2.5% and 2%, respectively.

**Fig. 7:**
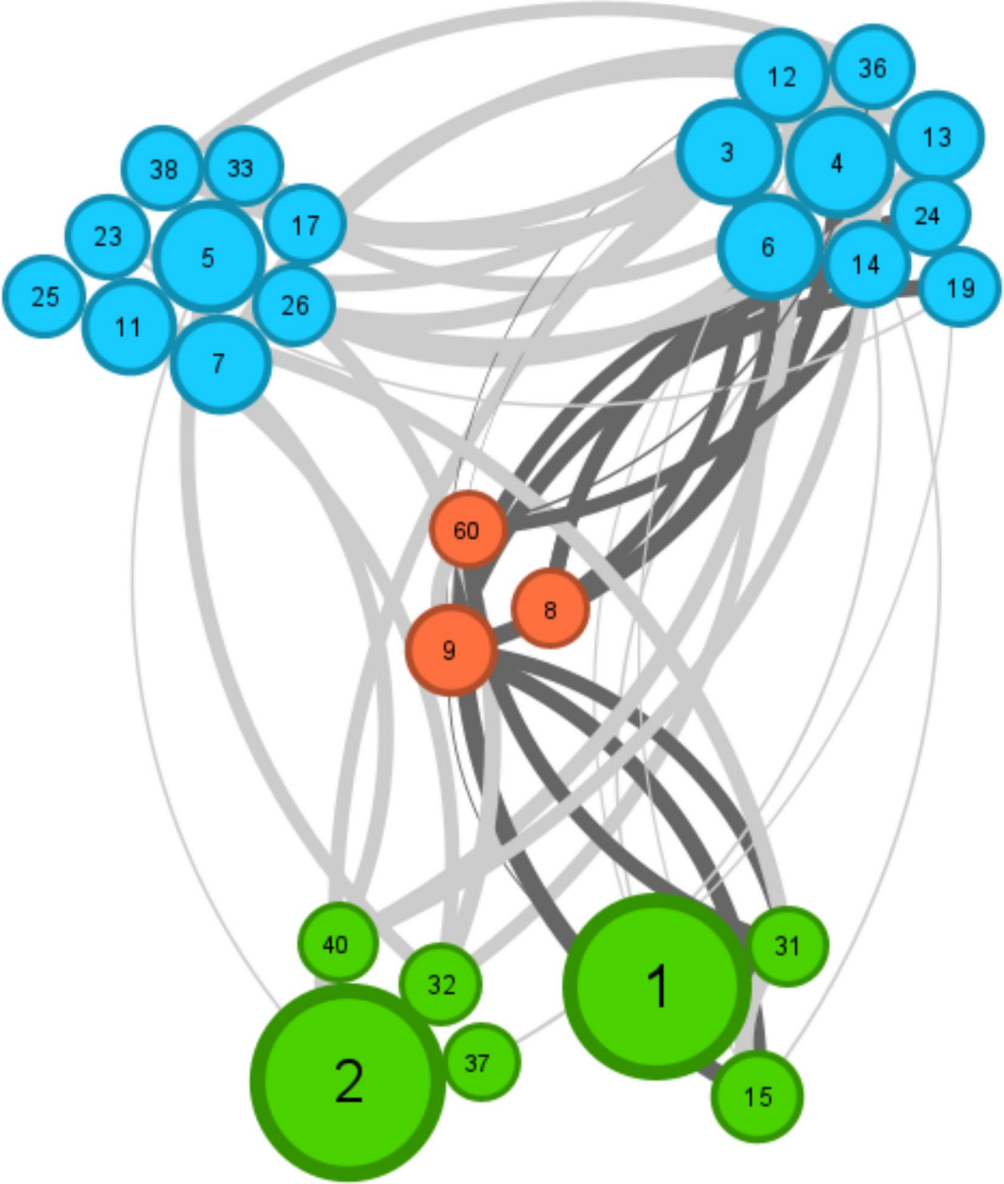
Identification of host-parasite interactions. Correlation network of the most abundant chytrid, diatom and green algae OTUs. Only significant (*p* < 0.05) correlations with correlation value > 0.5 are shown. Node labels represent OTU IDs. Node size represents the OTU abundance and nodes are colored by taxonomy classification: chytrids - red, diatoms - blue, green algae - green. Edge thickness correlates with correlation coefficient values. Edges highlighted in black represent correlations with chytrid OTUs.

Correlation analysis aimed to determine the distribution of chytrids in association with potential microalgal hosts. This analysis included only correlations between OTUs within the SSU dataset. For better visualization, we included only those correlations that had a correlation value higher than 0.5 (Fig. 7). The most abundant chytrid OTUs (OTU008, OTU009 and OTU060) were positively correlated with *Navicula* (OTU003, OTU013, OTU026), *Nitzschia* (OTU024), *Diatoma* (OTU004), *Surirella* (OTU006) and *Staurosira* (OTU011). Negative correlation was observed only with *Licmophora* (OTU036). Eight out of twelve infected samples, based on microscopy observations, contained the above-mentioned chytrid OTUs and fungal infections were observed under the microscope on *Navicula*- and *Nitzschia*-like diatoms and *Surirella*. *Surirella* OTU006 was positively correlated only with chytrid OTU008 and infected *Surirella* cells were observed under the microscope in two samples, both of which contained OTU006 and OTU008. Moreover, chytrid OTUs were also recovered from samples in which no infection under the microscope was observed. Additionally, strong correlations were found between chytrid OTU060 and OTU009 and two Chlorophyta OTUs (OTU015 and OTU031), classified as unknown Ulvophyceae and Chlorophyceae, respectively.

## Discussion

In this study, we investigated fungal and wider eukaryotic benthic communities following the salinity gradient of glacial freshwater runoff, with a specific focus on fungi within Chytridiomycota and their potential hosts. Both eukaryotic and fungal richness and diversity decreased with increased salinity. These results are consistent with other studies that identified salinity as a significant environmental driver of fungal communities^37,44–47^. While we found salinity to be a key factor influencing these communities, particularly when compared to temperature and pH, there are additional parameters, such as turbidity, nutrient levels, or zooplankton grazing, that were not addressed in this study but could also play an important role in shaping fungal communities. Moreover, many fungal OTUs were shared between limnic, brackish and marine habitats indicating that fungi detected in marine habitats may originate from terrestrial sources and be dispersed via meltwater runoff to coastal areas of the fjord, where they are capable of surviving. Halotolerance of aquatic fungi has been observed across various ecosystems and may represent an adaptive trait enabling them to cope with extreme environmental conditions, such as fluctuations in salinity, which is particularly important for fungi in the thawing Arctic^48^. The underlying physiological and biochemical mechanisms include, for example, the capability for osmotic adaptation, i.e. the intracellular control of inorganic and organic osmolyte concentrations^49^. In Kongsfjorden, a fjord influenced by the presence of four tidewater glaciers, benthic communities are particularly confronted with influxes from the glaciers and terrestrial melt freshwater that create steep turbidity, nutrient and salinity gradients along the length of this fjord. Especially during summer months, when the influx of meltwater is at its peak.

We reported the presence of Chytridiomycota in all sampled water types, with the highest abundance in marine samples. Previous studies from the Arctic have shown the affinity of chytrids for low salinity habitats^34^, predominantly melting sea-ice^37,39^ and glaciers^42,43^. As previously mentioned, coastal areas are highly affected by the inputs of glacier meltwater runoff that carries along sediment particles. Hasset and Gradinger^41^ reported a high representation of chytrid infections in seafloor sediments during periods of increased sea-ice melt, suggesting that sea-ice organisms may settle on the seafloor via sedimentation. Nevertheless, marine sediments of shallow zones may be advantageous for fungal lifestyle as they provide larger substrates for attachment (e.g. autochthonous and allochthonous organic material in case of saprotrophic chytrids). In addition, spread of chytrid infections might be easier in biofilms of benthic habitats due to the higher cell density of a host population compared to the water column. Furthermore, we identified a chytrid OTU that was specific only for marine habitat and it was recovered from the site that is less impacted by glacier meltwater input. This, together with the overall high abundances of chytrid OTUs in marine habitats, may suggest that some chytrids have either evolutionarily adapted to life in high salinity conditions or they proliferate in these conditions due to lower competition. Moreover, by placing our sequences in a phylogenetic tree, we demonstrated that our study captured a high diversity of chytrids in Arctic benthic habitats. This approach provided a greater insight into the previously unknown proportion of our chytrid sequences, but also emphasized that Arctic regions are still poorly studied and existing databases are still very limited. The majority of OTUs recovered in this study formed single branches within Chytridiomycota family clades and were more closely related to each other than the reference sequences. This particular pattern may suggest cold adaptation at the molecular level of chytrids in polar regions. It has been suggested that zoosporic fungi may employ different physiological strategies (e.g. resting stages) to survive a wide range of environmental conditions^50^, but molecular mechanisms still remain unknown.

Correlation analysis, coupled with microscopy observations, provided more detailed insights into the occurrence of putative parasitic chytrids and host-parasite interactions. We identified strong positive correlations between the most abundant chytrid OTUs and diatoms of the genera *Surirella*, *Navicula*, *Nitzschia* and *Staurosira*. These interactions were confirmed with a double-staining visualization method for fungal sporangia. We must consider that correlation analysis was done only within the SSU dataset, due to the compositional nature of two separate datasets, and it does not capture all sequence data obtained in this study, in particular, the fungal-specific LSU dataset. Therefore, some correlations were not detected in samples with fungal sporangia, although the highest abundance of a single chytrid OTU of the LSU dataset in these samples ranged from 45 to 73%. Previous studies conducted in the Arctic and sub-Arctic area have identified *Navicula* and *Nitzschia* as preferential hosts for chytrid parasites^37,38,41,51^, while evidence of *Surirella* as a host for parasitic chytrids was so far only presented by Friedmann^52^ who reported on chytrids infecting *Surirella* cells in a small Austrian stream near Vienna. In the present study, we report the first observations of fungal sporangia on *Surirella* diatom in sediments of the high Arctic region. In addition, we observed fungal sporangia on green algae filaments (identified as *Ulothrix*, based on microscopic observations) which were specific for marine habitats. In a recent review, Fredriksen et al.^53^ reported 5 *Ulothrix* species for Kongsfjorden, which form shiny, green mats of small unbranched filaments adherent to hard substrata or sediments in the intertidal zone. Chytrids are known to parasitize filamentous cyanobacteria^54,55^, or filaments of freshwater yellow-green algae *Tribonema*^56^, but we found no reports on infections of members of the Ulvophyceae, which dominated in our marine samples and in which fungal sporangia were observed. Given the saprotrophic lifestyle of chytrids, our observations must be interpreted cautiously. DNA databases for parasitic fungi are limited, making it difficult to accurately determine species function through environmental DNA alone. Specifically, for pennate diatoms, we predominantly observed fungal sporangia on dead, decolorized cells with depleted chloroplasts. This observation may indicate a saprotrophic lifestyle. However, parasitism cannot be entirely ruled out, as the empty sporangium attached to the diatoms could indicate the terminal stage of an infection. In addition, in all cases of putative chytrid infections, sporangia were detected on single, most dominant taxa in the sample. Some chytrid OTUs seemed to have a high affinity for one single host. For instance, although chytrid OTU060 showed positive correlations with both *Navicula* and Ulvophyceae, sporangia were observed solely on *Navicula*-like cells, despite the presence of Ulvophyceae filaments in the sample. Another example is chytrid OTU008, primarily found in samples with infected *Surirella* cells. Conversely, no infections were observed in samples containing this chytrid but lacking *Surirella*, instead they contained other pennate diatoms. Interestingly, Friedmann^52^ reported similar observations for *Chytridium surirellae*, which infected only *Surirella ovata*, despite the presence of other species of this taxon, or other pennate diatoms. Although it has been shown that chytrids can exhibit different infection strategies, ranging from specialists to more generalists, it is difficult to draw any strong conclusions based on our observations. In addition, a big proportion of our Chytridiomycota sequences were classified only to the Order level, indicating that high Arctic regions are still mainly unexplored and more detailed studies, including single cell-sequencing of host-parasite pairs, culturing and subsequent cross-infection experiments are clearly needed.

In summary, the data presented in this study provide evidence of salinity being a main abiotic factor in shaping microphytobenthic communities in Arctic coastal waters. Pronounced seasonality in physical drivers has been a dominant concept guiding the current understanding of the functioning of Arctic ecosystems. Considering that coastal waters are under constant influence of inputs of glacier meltwater runoff, and which harbor organisms that are key components in polar food webs, understanding their response to extreme fluctuations in abiotic factors is crucial, especially in the light of global change. As a main objective of our study, we reported on a high prevalence and diversity of chytrid fungi in Arctic benthic habitats, along with the evidence of their parasitic and/or saprotrophic lifestyle. Given the important roles chytrids play in regulating host population dynamics and in carbon and nutrient cycling, the observed shifts in microphytobenthic communities due to meltwater influxes could have far-reaching consequences for polar aquatic food webs. Ultimately, the relevance of fungal infections in Arctic ecology remains largely unknown. Nonetheless, our findings provide a baseline for future studies and underscore the necessity of studying these relationships to gain a comprehensive understanding of polar ecosystems.

## Methods

### Sites and sample collection

The study was conducted at the French-German Arctic Research Base AWIPEV in Ny-Ålesund, which is located on the southern shore of Kongsfjorden (78°55’N, 11°55’E). Kongsfjorden is an open, 26 km long fjord situated on the northwest part of Spitsbergen Island in the Svalbard archipelago. The inner part of the fjord is influenced by four tidal glaciers whose influxes, particularly during summer, create steep environmental gradients^57^. Sampling took place during northern polar summer, from August to September 2022 in several locations along the fjord, including Blomstrand and Prince Heinrich islands (Fig. 1). The sampling was done following a salinity gradient of the glacier meltwater runoff and it included marine shallow coastal areas (0–2 m depth) as well as freshwater ponds and lake shores and streams of brackish meltwater runoff. Muddy and sandy areas were sampled using a Plexiglas sediment corer that was manually pushed into the sediment (5 cm depth) and the upper 5 mm of the collected sediment layer, with the highest abundance of microalgae, was subsampled with a spatula. At several sites it was not possible to use a sediment corer due to the bigger grain size of the substrate. As such, the samples were collected using a plastic syringe and spatula. Areas dominated by rocky substrates were sampled by scratching off the biofilm surface of at least four stones with a sterile knife. Two samples were taken at each site: (i) for DNA analysis, samples were fixed with 70% ethanol (final concentration) and kept at − 20 °C until further processing; (ii) for microscopy, samples were fixed with Lugol’s Iodine solution and kept at 4 °C. Parameters such as temperature, pH, conductivity and salinity were recorded accurately at the time of sampling using a WTW Multiline P4 handheld probe and are shown in Supplementary Table 1.

### Epifluorescence microscopy

Lugol fixed sediment samples were diluted in 2 mL sterilized MiliQ water and hand-shaken to detach any epipsammic diatom taxa from the sediment particles. To visualize chytrid sporangia, samples were stained using a dual staining protocol with Calcofluor White (CFW) and Wheat Germ Agglutinin, conjugated to Alexa Fluor 488 (WGA)^58^. Cells were counted under an inverted microscope (Nikon eclipse Ti2, 400X, fluorescence channels CFW: 387/11 nm excitation and 442/46 nm emission, WGA: 482/35 nm excitation and 536/40 nm emission), according to the Utermöhl method. At least 300 microalgal cells were counted in each sample, if possible. In case of low microalgal abundance, the entire Utermöhl chamber was screened. The infection prevalence was calculated by dividing the number of infected host cells by the total number of host cells.

### DNA extraction and sequencing

DNA from ethanol fixed samples was extracted according to a modified protocol by^59^, as described in^44^. PCR, library preparation and sequencing were performed by Rush University Medical Center (Chicago, Illinois, USA). In order to study fungal diversity, D1 region of the 28S LSU rRNA gene was amplified using primers ITS4ngsF (5’-GCATATCAATAAGCGSAGGAA-3’) and LF402R (5’-TTCCCTTTYARCAATTTCAC-3’) (modified after^60^). To provide additional data on potential hosts for fungal parasites, the wider eukaryotic community was characterized by amplification of the V1-3 region of the 18S SSU rRNA gene using universal primer 27F (5’-GAAACTGCGAATGGCTC-3’) coupled with eukaryote-specific primer EK516R (5’-ACCAGACTTGCCCTCC-3’). Amplification was followed by library preparation and sequencing (2 × 300 bp) on a MiSeq (Illumina) platform. Raw sequence data is available in the European Nucleotide Archive (ENA) under BioProject PRJEB77092.

### Bioinformatics analysis

Raw sequence data was processed in R (ver. 4.0.3) using the DADA2 package^61^. Primers were removed from demultiplexed reads using cutadapt ver. 3.5^62^. The primer-free sequences were then filtered and trimmed to remove low-quality sequences using parameters maxN = 0, maxEE = 3, truncQ = 2. Paired-end reads were merged and used to construct amplicon sequence variants (ASVs). Chimeric sequences were removed using removeBimeraDenovo function, resulting in a total of 9811 ASVs in the SSU dataset and 3061 ASVs in the LSU dataset. The taxonomy of SSU ASVs was assigned using SILVA SSU v. 138 database^63^ for identification of all eukaryotic taxa and LSU ASVs were run against SILVA LSU v. 138.1 database^63^ for identification of fungal taxa. ASVs were clustered into OTUs at 97% similarity threshold using DECIPHER package^64^, generating 4750 OTUs in SSU and 938 OTUs in LSU dataset. New taxa are set to the name of the most abundant taxon within each cluster. Prior to any downstream analysis, singletons and doubletons were removed from both datasets, as well as OTUs with less than 5 reads in 5% of the samples. This resulted in a total of 446 OTUs in SSU and 151 OTUs in the LSU dataset. Taxonomic affiliation of OTUs from the LSU dataset was further manually verified by searching the NCBI nt database using BLASTn. Final taxonomic affiliation was based on > 95% sequence similarity and > 97% query coverage.

Taxonomic affiliation was not always possible beyond the phylum level (e.g. Chytridiomycota). To better resolve the approximate taxonomic affiliation of these sequences, we selected the top 23 fungal OTUs within Chytridiomycota and Blastocladiomycota and placed them into the SILVA LSU 138.1 tree using the Alignment, Classification and Tree (ACT) Service (https://www.arb-silva.de/aligner/*)*^65^. We included neighbor sequences (max 10 sequences) with a min. identity of 60% ANI. Tree inference was performed using RAxML with GTR model and a gamma distribution of substitution rates across sites.

### Statistical analysis

Data processing, statistical analysis and visualizations were performed in R. Differences in community structure in respect to water types between samples were calculated using abundance-based Bray–Curtis dissimilarity measure, using *phyloseq* package^66^ and visualized through non-metric multidimensional scaling (NMDS) ordination. Analysis of similarities (ANOSIM) in the *vegan* package^67^ was used to assess whether community structures were significantly different across different water types. Statistical significance was calculated with a post hoc pairwise t-test using “pairwise adonis” function^68^ with 999 permutations. P-values of the pairwise t-test were adjusted with Bonferroni method. Simple linear regression with Pearson’s correlations was used to identify the main environmental factors influencing eukaryotic and fungal abundance and diversity. To calculate alpha diversity values, *microbiome* package was used^69^. Significance in alpha diversity between different sample groups was tested by Kruskal–Wallis test, followed by pairwise Wilcoxon rank sum tests with Benjamini–Hochberg adjusted P values. Graphical representation of results was realized using the package *ggplot2*^70^. Ternary plots were made using *ggtern* package^71^. Venn diagram of shared and unique chytrid OTUs in different sample groups was produced using *ggvenn* package. Correlation analysis was performed within the SSU dataset using FastSpar^72^, which implements the SparCC algorithm^73^ and addresses the challenges associated with compositional data. FastSpar was run using default parameters and p-values were calculated from 1000 bootstrap correlations. Only significant (*p* < 0.05) correlations were included in the subsequent analysis. To visualize the correlations, a matrix subset was built, including only Chytridimycota OTUs that had > 1% prevalence in at least one sample and/or > 10% prevalence across all samples, and Bacillariophyceae and Chlorophyta OTUs with > 10% prevalence in at least one sample and/or > 50% prevalence across all samples. Correlations were visualized as a network produced using Gephi 0.10.1 software^74^. Map of sampling sites was made in R using *PlotSvalbard* package^75^.

## Electronic supplementary material

Below is the link to the electronic supplementary material.

Supplementary Material 1

## Data Availability

Datasets generated in this study are available in the European Nucleotide Archive (ENA) under BioProject accession number PRJEB77092.
