# Supplementary material for: Chytrid fungi infecting Arctic microphytobenthic communities under varying salinity conditions
Source: Sci Rep. 2024 Oct 28;14:25821. doi: 10.1038/s41598-024-77202-2 (PMC11519490; doi:10.1038/s41598-024-77202-2)
Supplement: Supplementary file 1 — Supplementary Material 1 [file 41598_2024_77202_MOESM1_ESM.docx]

**Supplementary Material for:**

**Exploring the unseen: chytrid fungi in Arctic microphytobenthic communities**

Doris Ilicic^1^, Jason Woodhouse^1^, Ulf Karsten^2,5^, Katherina Schimani^3^, Jonas Zimmermann^3^, Hans-Peter Grossart^1,4*^

^1^Department of Experimental Limnology, Leibniz Institute of Freshwater Ecology and Inland Fisheries, Neuglobsow, Germany

^2^Institute of Biological Sciences, Department of Applied Ecology and Phycology, University of Rostock, Rostock, Germany

^3^Botanic Garden and Botanical Museum Berlin, Freie Universität Berlin, Berlin, Germany

^4^Institute of Biochemistry and Biology, University of Potsdam, Potsdam, Germany

^5^Interdisciplinary Faculty, Department of Maritime Systems, University of Rostock, Rostock, Germany

***Correspondence**: Hans-Peter Grossart, [hgrossart@igb-berlin.de](mailto:hgrossart@igb-berlin.de)

**Supplementary Figures:**


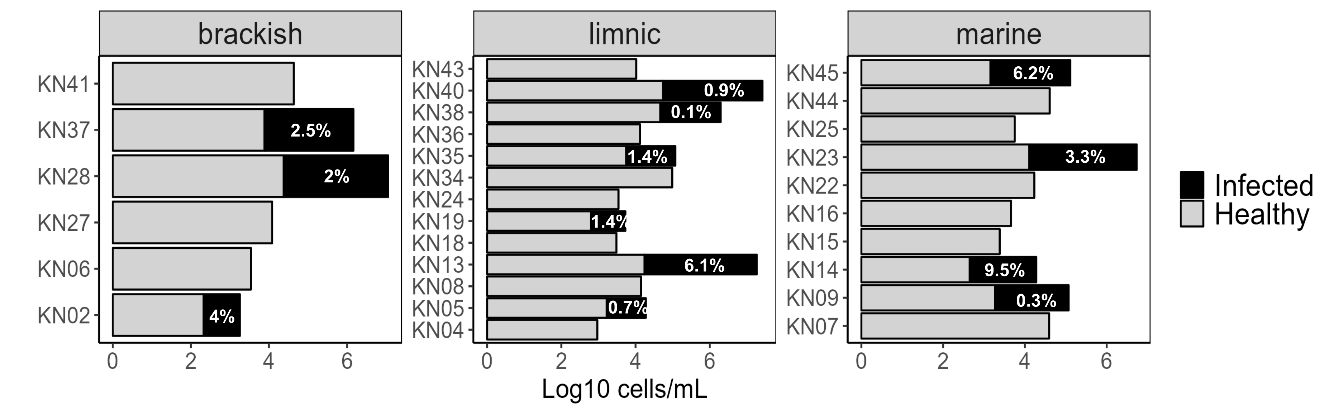


Supplementary Figure S1. Infection prevalence. Log-transformed abundances of non-infected and infected hosts per sample.


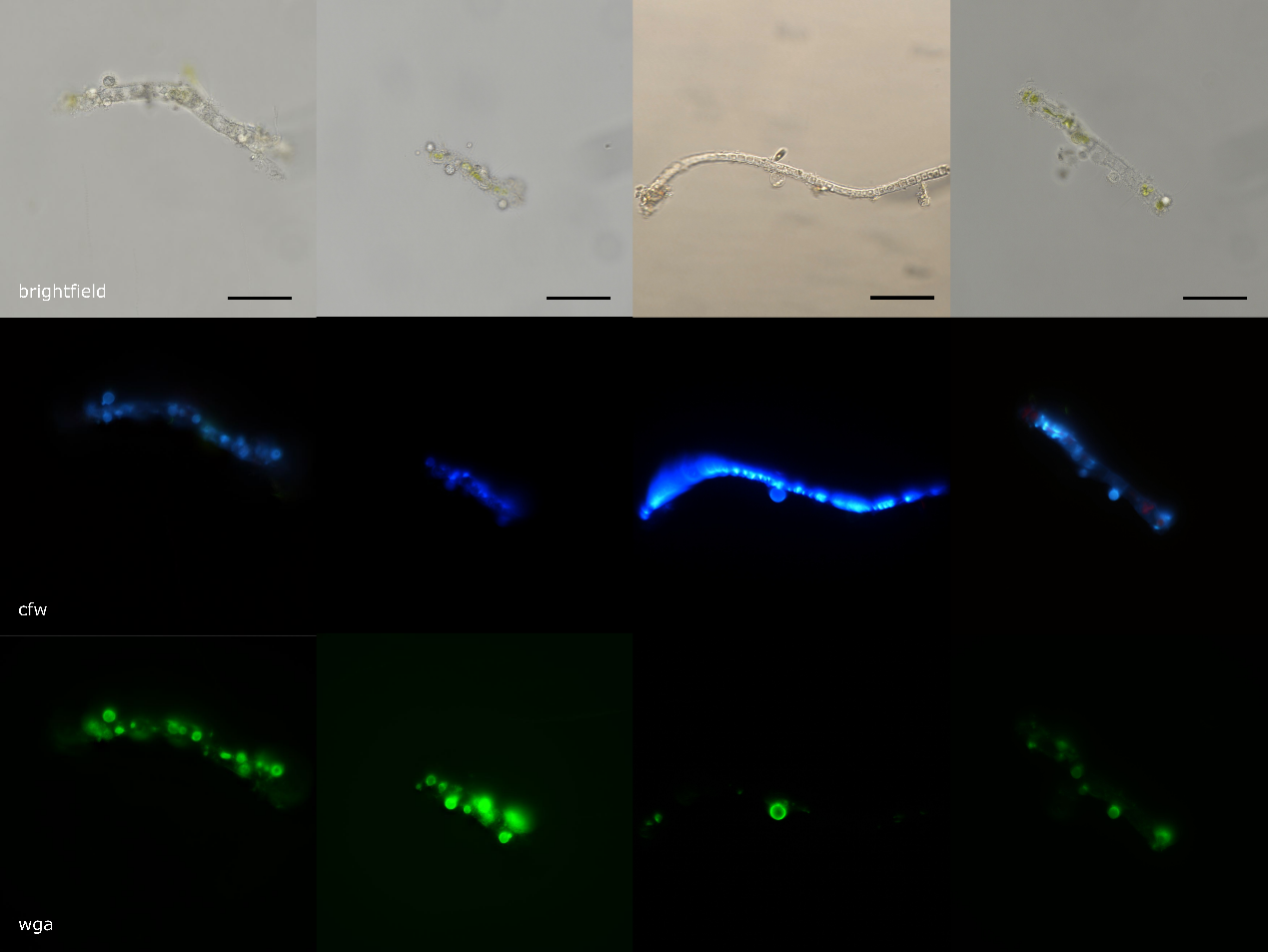


Supplementary Figure S2. Host-parasite interactions. Epifluoerescent microscopy of putative chytrid infections on green algae (putative *Ulothrix*) filaments. cfw=Calcoflour White, wga=Wheat Germ Agglutinin. Scale bar is 50 µm.


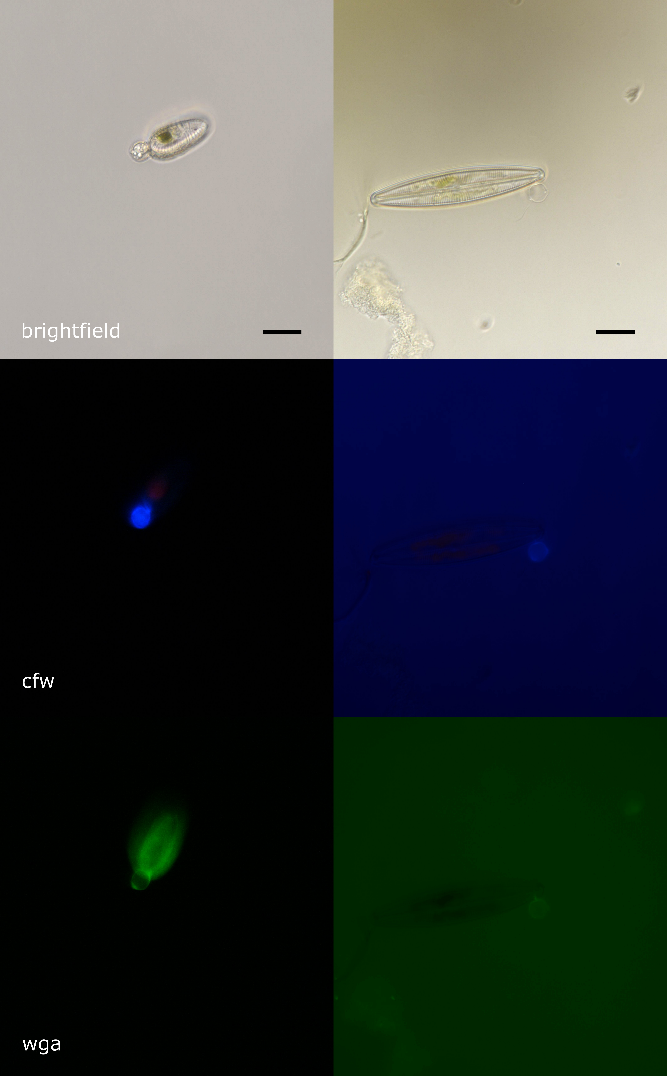


Supplementary Figure S3. Host-parasite interactions. Epifluoerescent microscopy of putative chytrid infections on benthic diatoms. cfw=Calcoflour White, wga=Wheat Germ Agglutinin. Scale bar is 20 µm.

**Supplementary Tables:**

Suplementary Table 1. Sampling sites characteristics and respective metadata.

| Sample | Latitude | Longitude | Water type | Temperature [°C] | pH | Conductivity [mS/cm] | Salinity [PSU] |
| --- | --- | --- | --- | --- | --- | --- | --- |
| KN01 | 78 55,23 | 11 57,15 | limnic | 8.1 | 8.3 | 0.43 | 0.27 |
| KN02 | 78 54,97 | 11 58,63 | brackish | 7.6 | 8.2 | 18.7 | 11.7 |
| KN04 | 78 54,84 | 12 01,79 | limnic | 3.9 | 8.0 | 0.4 | 0.2 |
| KN05 | 78 56,57 | 11 51,97 | limnic | 7.4 | 9.1 | 1.2 | 0.8 |
| KN06 | 78 56,56 | 11 52,18 | brackish | 3.7 | 8.0 | 33.7 | 21.2 |
| KN07 | 78 56,78 | 11 50,44 | marine | 6.5 | 8.0 | 54.2 | 34.1 |
| KN08 | 78 56,52 | 11 49,40 | limnic | 6.9 | 8.4 | 0.4 | 0.2 |
| KN09 | 78 55,62 | 11 54,37 | marine | 6.9 | 8.2 | 52.7 | 33.1 |
| KN13 | 78 55,55 | 11 54,32 | limnic | 6.2 | 8.0 | 0.5 | 0.3 |
| KN14 | 78 55,28 | 11 59,15 | marine | 5.5 | 8.1 | 52.1 | 32.8 |
| KN15 | 78 55,29 | 11 59,28 | marine | 5.4 | 8.1 | 51.6 | 32.6 |
| KN16 | 78 55,31 | 11 59,02 | marine | 4.9 | 8.0 | 51.7 | 32.7 |
| KN18 | 78 57,12 | 12 29,65 | limnic | 9.4 | 8.3 | 0.2 | 0.1 |
| KN19 | 78 57,23 | 12 29,72 | limnic | 9.1 | 8.1 | 0.4 | 0.2 |
| KN21 | 78 57,04 | 12 25,98 | brackish | 2.8 | 8.2 | 38.1 | 24.1 |
| KN22 | 78 57,74 | 12 02,92 | marine | 3.7 | 8.2 | 49.0 | 30.3 |
| KN23 | 78 57,72 | 12 03,14 | marine | 3.1 | 8.1 | 49.2 | 30.7 |
| KN24 | 78 57,98 | 12 03,43 | limnic | 4.6 | 7.8 | 0.4 | 0.2 |
| KN25 | 78 59,21 | 11 58,41 | marine | 4.8 | 8.1 | 48.8 | 30.5 |
| KN26 | 79 00,23 | 11 59,87 | marine | 3.3 | 8.1 | 44.2 | 27.3 |
| KN27 | 79 00,31 | 11 59,63 | brackish | 10.4 | 8.4 | 2.5 | 1.1 |
| KN28 | 78 59,64 | 12 06,13 | marine | 4.3 | 8.1 | 47.7 | 29.6 |
| KN31 | 78 53,43 | 12 21,13 | marine | 3.6 | 8.1 | 49.4 | 31.0 |
| KN34 | 78 57,80 | 11 37,71 | limnic | - | 8.0 | 0.4 | 0.2 |
| KN35 | 78 57,52 | 11 41,74 | limnic | - | 8.2 | 0.3 | 0.2 |
| KN36 | 79 01,41 | 12 07,44 | limnic | - | 8.2 | 0.1 | 0.1 |
| KN37 | 79 01,36 | 12 07,32 | marine | - | 8.0 | 36.3 | 22.8 |
| KN38 | 78 56,79 | 11 46,37 | limnic | - | 8.2 | 0.3 | 0.0 |
| KN39 | 78 55,65 | 11 55,94 | marine | 5.3 | 8.2 | 52 | 32.9 |
| KN40 | 78 55,15 | 11 57,89 | limnic | - | 8.4 | 0.9 | 0.2 |
| KN41 | 78 54,97 | 11 58,67 | brackish | - | 8.1 | 29.3 | 17.8 |
| KN43 | 78 54,88 | 12 03,84 | limnic | 4.7 | 8.2 | 0.5 | 0 |
| KN44 | 78 55,60 | 11 56,45 | marine | 5.3 | 8.1 | 51.7 | 32.6 |
| KN45 | 78 55,63 | 11 56,30 | marine | 5.3 | 8.1 | 51.7 | 32.6 |

Supplementary Table 2. Pearson's correlation analysis of environmental parameters and eukaryotic and fungal species’ richness and diversity

|  | 18S | | LSU | |
| --- | --- | --- | --- | --- |
| Variable | Richness | Shannon index (*H’*) | Richness | Shannon index (*H’*) |
| Temperature | 0.46* | 0.29 | 0.29 | 0.33 |
| Salinity | -0.46** | -0.35* | -0.32 | -0.38* |

Notes: * Correlation is significant at the 0.05 level; ** Correlation is significant at the 0.01 level

Supplementary Table 3. Model summary of simple linear regression analysis

|  |  | Coefficients | | Std. Error | t-value | p-value | | Significance |
| --- | --- | --- | --- | --- | --- | --- | --- | --- |
| Richness – 18S | Intercept | 4.444 | | 26.353 | 0.169 | 0.8675 | |  |
|  | Temperature | 11.351 | | 4.397 | 2.581 | 0.0161 | | * |
|  | Multiple R-squared: 0.2105, Adjusted R-squared: 0.1789 | | | | | | | |
|  | F-statistic: 6.664 on 1 and 25 DF, p-value: 0.01609 | | | | | | | |
| Shannon – 18S | Intercept | 1.1008 | 0.7075 | | 1.556 | | 0.132 |  |
|  | Temperature | 0.1822 | 0.1181 | | 1.543 | | 0.135 |  |
|  | Multiple R-squared: 0.08698, Adjusted R-squared: 0.05046 | | | | | | | |
|  | F-statistic: 2.382 on 1 and 25 DF, p-value: 0.1353 | | | | | | | |
| Richness - LSU | Intercept | 11.227 | | 6.084 | 1.845 | 0.0769 | | . |
|  | Temperature | 1.555 | | 1.015 | 1.532 | 0.1380 | |  |
|  | Multiple R-squared: 0.2783, Adjusted R-squared: 0.1842 | | | | | | | |
|  | F-statistic: 2.957 on 3 and 23 DF, p-value: 0.0 | | | | | | | |
| Shannon – LSU | Intercept | 0.97412 | | 0.42033 | 2.317 | 0.0290 | | * |
|  | Temperature | 0.12284 | | 0.07014 | 1.751 | 0.0921 | | . |
|  | Multiple R-squared: 0.1093, Adjusted R-squared: 0.07366 | | | | | | | |
|  | F-statistic: 3.067 on 1 and 25 DF, p-value: 0.09214 | | | | | | | |

Notes: Signif. codes: 0 ‘***’ 0.001 ‘**’ 0.01 ‘*’ 0.05 ‘.’ 0.1 ‘ ’ 1
